# Detection of Tannery Effluents Induced DNA Damage in Mung Bean by Use of Random Amplified Polymorphic DNA Markers

**DOI:** 10.1155/2014/727623

**Published:** 2014-03-10

**Authors:** Abhay Raj, Sharad Kumar, Izharul Haq, Mahadeo Kumar

**Affiliations:** ^1^Environmental Microbiology Section, CSIR-Indian Institute of Toxicology Research, M.G. Marg, Post Box No. 80, Lucknow 226 001, India; ^2^Animal Facility, CSIR-Indian Institute of Toxicology Research, M.G. Marg, Post Box No. 80, Lucknow 226 001, India

## Abstract

Common effluent treatment plant (CETP) is employed for treatment of tannery effluent. However, the performance of CETP for reducing the genotoxic substances from the raw effluent is not known. In this study, phytotoxic and genotoxic effects of tannery effluents were investigated in mung bean (*Vigna radiata* (L.) Wilczek). For this purpose, untreated and treated tannery effluents were collected from CETP Unnao (UP), India. Seeds of mung bean were grown in soil irrigated with various concentrations of tannery effluents (0, 25, 50, 75, and 100%) for 15 days. Inhibition of seed germination was 90% by 25% untreated effluent and 75% treated effluent, compared to the control. Plant growth was inhibited by 51% and 41% when irrigated with untreated and treated effluents at 25% concentration. RAPD technique was used to evaluate the genotoxic effect of tannery effluents (untreated and treated) irrigation on the mung bean. The RAPD profiles obtained showed that both untreated and treated were having genotoxic effects on mung bean plants. This was discernible with appearance/disappearance of bands in the treatments compared with control plants. A total of 87 RAPD bands were obtained using eight primers and 42 (48%) of these showed polymorphism. Irrigating plants with untreated effluent caused 12 new bands to appear and 18 to disappear. Treated effluent caused 8 new bands and the loss of 15 bands. The genetic distances shown on the dendrogram revealed that control plants and those irrigated with treated effluent were clustered in one group (joined at distance of 0.28), whereas those irrigated with untreated effluent were separated in another cluster at larger distance (joined at distance of 0.42). This indicates that treated effluent is less genotoxic than the untreated. Nei's genetic similarity indices calculated between the treatments and the control plants showed that the control and the plants irrigated with treated tannery effluent had a similarity index of 0.75, the control and plants irrigated with untreated 0.65, and between the treatments 0.68. We conclude that both untreated and treated effluents contain genotoxic substances that caused DNA damage to mung beans. CETP Unnao removes some, but not all, genotoxic substances from tannery effluent. Consequently, use of both untreated and treated wastewater for irrigation poses health hazard to human and the environment.

## 1. Introduction

Large amount of water and chemicals like chromium, sodium chloride, sulphate, calcium salts, ammonium salts, sodium sulphide, alkali, acids, fat, liquor, and organic dyes are used in tannery during processing of raw hide/skin into leather. The resultant effluents contain excess amount of dissolved salts: chloride, sulphide, chromium, and high BOD and COD in the effluent [1]. India is the third largest producer of leather in the world having about 3000 tanneries with annual processing capacity of 0.7 million tonnes of hides and skin [2]. Nearly 80% of the industries in India are engaged in the chrome-tanning process. Residual chromium (trivalent, Cr III and hexavalent, Cr VI) thus is discharged in solid or liquid effluents. Soluble Cr VI is extremely toxic and shows mutagenic and carcinogenic effects on biological systems due to its strong oxidizing nature. Cr III is less toxic and bioavailable than Cr VI, as it readily forms insoluble oxides and hydroxides above pH 5. In case of plants, tannery effluent is known to inhibit seed germination and root growth [3, 4].

Higher plant provides a useful genetic system for screening and monitoring of environmental pollutants [5]. They are good indicators of cytogenetic and mutagenic effect which can be applied both indoors and outdoors. Several studies have used the chromosome aberration, micronucleus, or comet assay to measure the effects of tannery effluent on plants [2, 6]. The micronucleus test in organism assays is not a sensitive method for determining pollution levels of heavy metals in terrestrial ecosystems, and another difficulty linked to the comet assay is the necessity to experiment on isolated cells and their inability to provide information on the effects of toxic metals at the DNA level [7]. Advantage of measuring effect of genotoxic chemicals directly on DNA is mainly related to the sensitivity and short time response. Recently, advances in molecular biology have led to development of a number of selective and sensitive assays for DNA analysis in ecogenotoxicology. DNA based techniques (RFLP, QTL, RAPD, AFLP, SSR, and VNTR) are used to evaluate the variation at the DNA sequence level. Random amplified polymorphic DNA (RAPD) of these techniques is a PCR-based method that amplifies random DNA fragments with the use of single short primers of arbitrary nucleotide sequence under low annealing conditions. RAPD can be used to detect genotoxicity and differences can be clearly shown when comparing DNA fingerprint from untreated and treated individuals to genotoxic agents [8–14].

Unnao district (U.P.), India, is one of the major industrial areas especially for leather tannery industry. Most of the tanneries in this area are in the small scale and cannot afford expensive treatment plant on their own. Therefore, to mitigate the pollution from tanneries, a common effluent treatment plant (CETP) was established in 1996 having treating capacity of 2.15 million liters per day (MLD). This is an activated sludge process-based plant which receives 1.9 million liters per day (MLD) of tannery wastewater from a cluster of 21 tanneries processing approximately 47.5 tons average daily raw hides. The treated effluent from CETP is finally discharged in the Ganga River through drain. This wastewater is used for irrigation purposes in agricultural field on both sides of drain before entering in the Ganga River. Ramteke et al. [15] assessed the efficiency of CETP and found a significant reduction in COD and BOD levels during the course of treatment in CETP. However, the impact of CETP treatment for reducing genotoxic substances from raw effluent is not known. Therefore, in the present study, the impact of CETP on genotoxic activity of tannery effluent was assessed by plant genotoxicity analysis. The present study uses RAPD technology to evaluate the genotoxic effects in mung bean plant irrigated with untreated and treated tannery effluents.

## 2. Materials and Methods

### 2.1. Effluent Samples and Physicochemical Analysis

The untreated (UT) and treated (T) effluents were collected from inlet and outlet points, respectively, from common effluent treatment plant (CETP) Unnao (26.48°N, 80.43°), U.P. state, India, during the month of February 2013. Samples were taken in 5-litre Jerry cane and transported to laboratory for analysis. The effluents were filtered through Whatman filter paper number 1 to remove suspended particles and stored at 4°C till completion of the analysis. The following parameters were determined, based on standard methods [16]: pH, total dissolved solids (TDS), total hardness, total alkalinity, chemical oxygen demand (COD), biochemical oxygen demand (BOD), and total chromium (total Cr). Total Cr was analyzed with atomic absorption spectrometer (Perkin Elmer model, A. Analyst-300) after digestion of samples (100 mL) in digestion mixture of (5 : 1) of nitric-perchloric acid [16]. Electrical conductivity was determined by conductivity meter (Thermo Orion, model-162A, USA).

### 2.2. Plant Growth Experiment

The mung bean,* Vigna radiata* L. Wilczek (var. K-851), a most important pulse crops plant grown in India especially in Indo-Gangetic plains, was used in this study. Bean is a diploid (2*n* = 22) and it has been used in physiological, biochemical, and molecular analyses of toxicology [11, 13]. The selected mung bean seeds were surface-sterilized with 75% (v/v) ethanol for 5 min, followed by 10% (v/v) sodium hypochlorite for 10 min, and washed thoroughly with distilled water [13]. Seed germination and plant growth were studied in plastic beakers (size, 75 mm × 90 mm) filled with 250 g of oven-dried powdered garden soil. Four treatment concentrations, namely, 25, 50, 75, and 100% v/v prepared by diluting tannery effluents with distilled water, were used to irrigate pots. Each treatment was run in triplicate with 10 seeds/beaker. Beakers irrigated with only distilled water were taken as control. Seeds were allowed to germinate and grow at room temperature. Proper moisture was maintained in pots using equal volume (50 mL) of test solutions after 3 days of time interval. After 15 days, shoots from plants were taken from each group and pooled for DNA extraction. Seed germination percentages and plant growth were also determined from the same experimental slots.

### 2.3. DNA Extraction

Shoots were thoroughly washed with double distilled water and DNA was extracted from the shoots using the GeneiPure Plant Genomic DNA Purification Kit (Merck, India) following manufacturer's procedure as follows: about 0.3 g of fresh plant shoots was homogenized in a cold mortar with 400 *μ*L of lysis buffer and lysate was transferred into the 1.5 mL microcentrifuge tubes. Ten *μ*L RNase A solution (10 mg/L) was added to the lysates and mixed well before incubation at 65°C for 30 min. It was then added with 75 *μ*L of precipitation buffer, mixed thoroughly, and incubated for 5 min on ice. The lysate was transferred into GenePure column (violet ring) and centrifuged for 2 min at 11000 rpm to remove cellular debris. The obtained flow-through was mixed with 450 *μ*L binding buffer, loaded onto the GenePure column (green ring), and centrifuged for 1 min at 11000 rpm. Flow-through was discarded and column was washed with washing buffer 1 (400 *μ*L) and subsequently with washing buffer 2 (700 *μ*L) to remove the residual debris. The bound DNA was eluted with 40 *μ*L of prewarmed (70°C) elution buffer and stored at −20°C. DNA yield was calculated by spectrophotometer (Techcomp, Korea) at 260 nm. The index of DNA purity (OD260/280) was found to be 1.80. The integrity of the extracted DNA was analyzed by electrophoresis through 0.8% agarose gel, visualized under UV transilluminator, and photographed and recorded using the Gel Documentation system (G:BOX, Syngene, U.K.).

### 2.4. RAPD Analysis

Ten decamer plant specific primers in this study (RPi-C1 to RPi-C10, cat number 610692700101730) purchased from Merck, India, were used. Sequences (5′→3′) from 1 to 10 utilized are AAAGCTGCGG (RPi-C1); AACGCGTCGG (RPi-C2); AAGCGACCTG (RPi-C3); AATCGCGCTG (RPi-C4); AATCGGGCTG (RPi-C5); ACACACGCTG (RPi-C6); ACATCGCCCA (RPi-C7); ACCACCCACC (RPi-C8); ACCGCCTATG (RPi-C9); ACGATGAGCG (RPi-C10), respectively. PCR reactions were performed in reaction mixture of 25 *μ*L containing 12.5 *μ*L of 2X GeNei HotStart PCR Master Mix (Merck), 1 *μ*L template DNA (25 ng), 1 *μ*L (25 pmol) primer, and 10.5 *μ*L nuclease-free water (Merck). Amplification was performed at following PCR cycle program: initial denaturation for 5 min at 94°C followed by 38 cycles each consisting of 1 min at 94°C (denaturing), 1 min at 36°C (annealing), 1 min at 72°C (extension), and followed by 1 cycle for 5 min at 72°C (final extension step). Before running the RAPD analysis, optimization of PCR conditions was performed to check reproducibility and consistency. Negative controls with water, replacing the template DNA, were always included to monitor the contamination. RAPD amplified products were electrophoresed on 1.8% agarose gel stained with ethidium bromide in 1x TAE buffer. GeNei low range DNA ruler plus (100–3000 bp) was used as molecular weight DNA standard. The size of each amplified product was approximated using GeneTools gel image analyzer software available with the Gel Documentation system.

### 2.5. Band Scoring and Data Analysis

PCR products were scored for appearance and disappearance of band for each primer analyzed. Only reproducible bands were scored. Scoring and matching of bands in different gels were done by Genesnap and Genetool software (Syngene, UK) along with the GeNei low range DNA rulers (100 bp and 3000 bp). The presence and absence of RAPD band were recorded as “1” and “0,” respectively. The binary coded characters (1, 0) were used for the genetic analysis. The scores obtained using all primers in the RAPD analysis were then pooled for constructing a single data matrix. The genetic similarity among the plants irrigated with untreated effluent, treated effluent, and water (control) was estimated from Nei's unbiased measure [17] in POPGENE version 1.31 software. Cluster analysis was performed and a dendrogram generated using the unweighted pair group method with the arithmetic means (UPGMA) algorithm of POPGENE [18].

## 3. Results and Discussion

### 3.1. Characteristics of Tannery Effluents

Physicochemical analysis of both effluents is indicated by high pH, EC, TDS, BOD, COD, and chromium, which is a clear reflection of the presence of high amount of organic and inorganic compounds (Table 1). However, treated effluent had comparatively low amount of pollutants due to treatment effect of CETP. The values of TDS, COD, and BOD recorded in the treated effluent were 6.7, 5.1, and 21.7 times higher than the standard limits as recommended by ISI [19] for industrial wastewater discharge. The results of comparative physicochemical analysis indicated that CETP treatment removes COD and BOD by 55 and 53%, respectively, but not EC, TDS, and chromium significantly. The high EC and TDS are due to use of large quantities of salts. Salts are very much soluble in water and chemically stable under aquatic conditions, making it effectively impossible to remove them easily from effluent. Chromium concentration in treated effluent was 3.81 mg/L which is higher than the discharge limit of 2 mg/L [19]. Continuous discharge of chromium in effluent even at low concentration can cause toxic effect on aquatic life by disrupting food chain [20].

### 3.2. Effect of Tannery Effluents on Germination and Growth of Mung Bean Seeds

**Figure 1:**
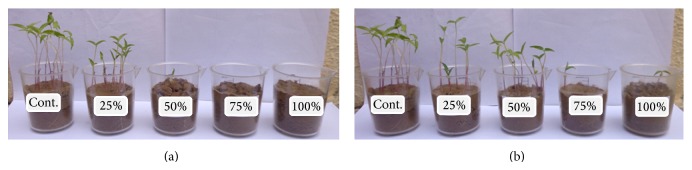
Effect of untreated (a) and treated (b) tannery effluents on seedling growth of mung bean at different concentrations.

Figure 1 shows an example of the germination and growth of mung bean seeds in plastic pots and recorded data are given in Table 2. When irrigated with untreated effluent, germination was inhibited by 90% at 50% concentration and prevented by 75% concentration. When irrigated with treated effluent, 90% germination was inhibited at both 75 and 100% concentrations. It is clear that untreated effluent above 25% and treated effluent above 50% had toxic effect on seed germination (Figure 1). This indicated that untreated tannery effluent was more toxic than the treated one. The total length of plant reduced with increasing effluents concentration as compared to control and it was more affected by untreated than the treated with the same concentrations. However, the root length of plant irrigated with 25% treated effluent was increased by 3.0 cm as compared to control 2.4 cm (Table 2). On the other hand, shoot length of plants showed a decreasing trend with increasing concentration of both untreated and treated tannery effluents. Thus, the reduction in seed germination percentage and plant growth at higher concentration of tannery effluents may be attributed to high salts and various ingredients of effluent, like sulphide or chromium, as reported by several workers [3, 21, 22]. The salt content outside the seed causes less absorption of water by osmosis and inhibits the germination of seeds, while chromium in addition to germination also affects plant growth by inhibition of root cell division and elongation. On the other hand, excess amount of sulphide present in effluent may induce deficiency of cationic micronutrient such as Zn, Cu, Fe, and Mn. Significant reduction in plant growth was observed at 25%, and therefore plant from this concentration was used to extract DNA for RAPD analysis.

### 3.3. RAPD Profile of the Control and Treated Plants

RAPD technique has shown promising result in the detection of pollutant-induced DNA effects due to its rapidity, applicability to any organism (since no information on the nucleotide sequence, cell cycle, or chromosome complement is required), and sensitivity to detect a wide range of DNA damage and mutations [9–11]. However, RAPD is a qualitative method and the nature and amount of DNA effects can only be speculated. At the same time, this technique has been invariably used to detect DNA damage and mutation in plants induced by heavy metals [11, 13, 14] and wastewaters [23–25]. In this study, RAPD analysis was performed on three pooled genomic DNA extracted from shoots of 25% of untreated and treated and control plants after 15 days. The reason for pooling genomic DNA from three individuals was to avoid the intrapopulation genetic polymorphism potentially revealed by RAPD analysis [14]. Ten decamer plant specific primers (RPi-C1–RPi-C10) of 60–70% GC content were utilized for screening of the mung bean genome for changes. Among them, 8 primers (RPi-C1–RPi-C8) gave clear and stable bands. RAPD profiles generated by these primers revealed differences between control and treated plants with visible change in the number and size of amplified DNA fragments. A total of 87 RAPD bands were obtained by using 8 primers and 42 (48%) of these bands showed polymorphism. Other studies obtained similar percentage of polymorphic bands, 48% [11] and 46% [25]. However, some other studies [26] found much higher percentage (83.56%) of polymorphic bands. Further, all bands generated were ranged between 160 and 1114 bp. The band size obtained in this study is within the range of other studies. Enan [11] analyzed the genotoxic effect of heavy metals in kidney bean (*Phaseolus vulgaris*) using RAPD primers. RAPD profiles obtained from six primers exhibited bands between 200 and 1600 bp in length. Swaileh et al. [25] obtained bands ranging from 150 bp to 2000 bp from 15 primers used to detect the wastewater-induced genotoxicity in oat plants (*Avena sativa*).

**Figure 2:**
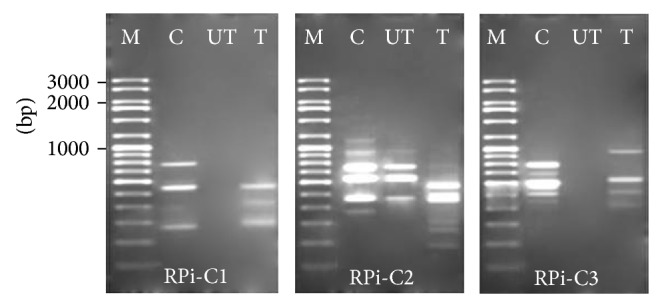
RAPD profiles generated by RPi-C1–RPi-C3 from mung bean plant irrigated with untreated (UT) and treated (T) tannery effluents. Lane C: control and Lane M: low range DNA rulers (100–3000 bp).

Figure 2 shows an example of the change in the banding patterns between control and treatments. The change in RAPD profile of the control plants and treatments was discernible from appearance/disappearance of some bands when treatments were compared with control (Table 3). Eight primers generated a total of 34 RAPD bands ranging from 160 to 1170 bp in control plants. All primers generated 4 RAPD bands with different size except RPi-C2, which gave 6 bands. Compared with the control plants, those irrigated with untreated effluent recorded the appearance of 12 new bands and disappearance of 18 bands. Plants irrigated with treated effluent yielded 8 new bands and 15 bands disappeared. The total number of bands that appeared and disappeared because of both treatments were 20 and 33, recpectively. This indicates that both untreated and treated effluent induced DNA change in plants. However, the number of bands that disappeared was higher in plants irrigated with untreated effluent as compared to those irrigated with treated effluent, which revealed that DNA damage was more severe in plants irrigated with untreated effluent. Enan [11] found that 22 new fragments appeared and 43 disappeared as result of using 350 mg/L heavy metals to irrigate kidney bean. Less band appearance/disappearance was observed when using 150 mg/L. Similarly, Cenkci et al. [13] found that 21 and 11 new bands appeared and 64 and 42 disappeared in root of* Phaseolus vulgaris* L. seedling exposed to Hg, Cr, and Zn at 350 and 150 mg/L, respectively.

The disappearance of control bands may be related to the DNA damage (e.g., single- and double-strand breaks, modified bases, abasic sites, oxidized bases, bulky adduct, and DNA-protein cross links), point mutations, and/or complex chromosomal rearrangements induced by genotoxins [10, 27]. When Taq DNA polymerase encounters a DNA adduct, there are number of possible outcomes including blockage, bypass, and the possible dissociation of the enzyme/adduct complex which will cause the loss of bands [28]. The appearance of new PCR products may reveal a change in some oligonucleotide priming sites due to mutations (new annealing event(s)), large deletions (bringing preexisting annealing site closer), and/or homologous combination (juxtaposing two sequences that match the sequence of primer) [9]. Atienzar et al. [29] reported that mutation can only be responsible for the appearance of new bands if they occur at same locus in a sufficient number of cells (a minimum of 10% of mutations may be required to get new PCR product visible in agarose) to be amplified by PCR. Briefly, new bands could be attributed to mutation, while the bands which disappeared could be attributed to DNA damage [28].

**Figure 3:**
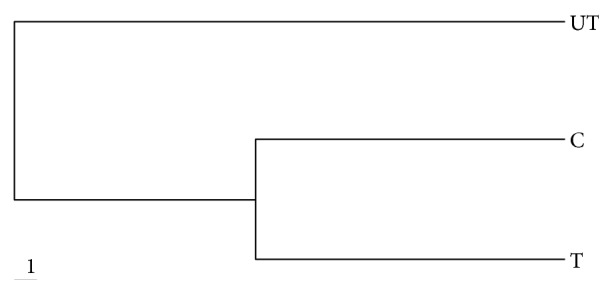
Dendrogram representing genetic distance among three samples of mung bean plants by UPGMA method based on RAPD analysis. C = control, T = treated, and UT = untreated.

The cluster analysis method is considered one of the most effective methods in numerical analysis regarding band scoring and analysis of RAPD finger printing [25]. It can calculate the distances between every pair of entities and then summarize the community data sets. In the present study, cluster analysis was done to estimate the level of DNA polymorphism between the control plants and those irrigated with untreated or treated tannery effluent. A dendrogram was constructed using distances matrix by using UPGMA method of phylip software (Figure 3). The control and treated samples were clustered in one group (join at distance of 0.28) and the untreated were separated in another cluster at larger distance (join at distance of 0.42). This result clearly showed that untreated effluent contains much more genotoxic substances than the treated one. Swaileh et al. [25] demonstrated that there is larger distance between control and raw wastewater as compared to between control and treated plants.

**Figure 4:**
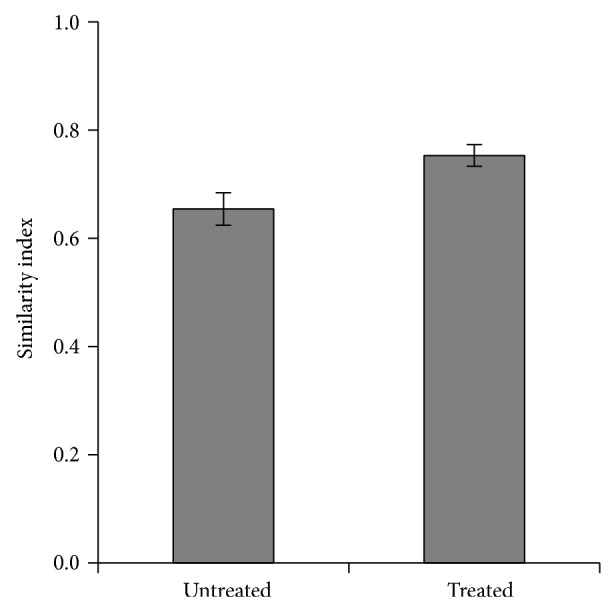
Genetic similarity between mung bean plants irrigated with untreated and treated tannery effluents and those irrigated with distilled water (control). Error bars represent ± standard deviation of the mean values from 8 primers.

Genetic diversity of population, that is, genetic similarity indices is an important parameters in RAPD analysis [27, 30]. Nei's genetic similarity indices, which measure the proportion of the shared fragments in the amplifications, were calculated and are shown in Figure 4. Mung bean plants irrigated with distilled water (control) and those irrigated with treated tannery effluent showed genetic similarity 75%. The plant irrigated with untreated tannery effluent showed clearly less genetic similarity (65%) to the control ones. In addition, plant irrigated with treated tannery effluent showed 68% similarity to those irrigated with untreated tannery effluent. This is in accordance with the results obtained from the cluster analysis, where the control and treated tannery effluent irrigated plants grouped in one cluster and the plants irrigated with untreated tannery effluent were grouped in a separate cluster joined at a larger distance.

The DNA damage in mung bean plants is possibly due to toxic effect of 4.48 and 3.81 mg/L chromium present in untreated and tannery effluent. Chromium (>2 ppm) has been reported to be inhibitory for plant growth. Among the different forms of chromium, hexavalent chromium (Cr. VI) is more toxic and carcinogenic due to its high solubility in water, rapid permeability through biological membrane, and subsequent interaction with intracellular proteins and nucleic acid [31]. Genotoxic effect of chromium in plants has been well documented; for instance, DNA damage due to exposure to chromium was observed in* Vicia faba* [32]. Labra et al. [33] found hypermethylation of DNA and increase in DNA polymorphism in* Brassica napus *in response to Cr (VI) exposure. Cr (VI) also induced genotoxicity detected by AFLP analysis in* Arabidopsis thaliana* L. [34]. Furthermore, Knasmüller et al. [35] compared Cr (VI) and Cr (III) with respect to their ability to induce micronucleus in* Tradescantia *and found that only in Cr (VI) exposed plants, there was an increase in micronucleus frequencies. Previous studies have showed that tannery effluent (12.5%) and Cr (4 mg/L) induced various chromosomal abnormalities in plant cells, thereby severely reducing mitotic index and root growth [2, 36]. To the best of our knowledge, there is no report available on plant DNA damage induced by tannery effluent using RAPD analysis. Hence, the finding of the present study increased our understanding on treatment efficiency of CETP and nature of wastewater, which is being discharged into the environment after treatment. In conclusion our data showed that both untreated and treated tannery effluents had genotoxic substances causing different levels of genotoxic effects. However, untreated effluent is more genotoxic than the treated one. Thus, from this study it is clear that CETP Unnao removes some, but not all, genotoxic substances found in untreated tannery effluent. Consequently, use of treated wastewater for irrigation poses health hazard to both human and animals of the environment.

## Figures and Tables

**Table 1:** Physicochemical characteristics of tannery effluents from CETP Unnao. Values are mean ± standard deviation (*n* = 3).

Parameter	Untreated	Treated	Reduction in pollutants (%)	ISI standard∗
pH	8.51 ± 0.4	8.28 ± 0.4	3	5.5–9.0
Electrical conductivity (mS/cm)	782 ± 1.1	761 ± 1.1	3	NS
Total dissolved solids (mg/L)	15200 ± 760	14100 ± 705	7	2100
Total hardness (mg/L)	1360 ± 68	940 ± 47	31	600
Total alkalinity (mg/L)	2400 ± 120	1900 ± 95	21	NS
Chemical oxygen demand (mg/L)	2848 ± 142	1280 ± 64	55	250
Biochemical oxygen demand (mg/L)	1375 ± 69	650 ± 33	53	30
Total chromium (mg/L)	4.48 ± 0.12	3.81 ± 0.12	3	2.0

^∗^ISI standards number 2490 (1974); NS: not specified.

**Table 2:** Effect of untreated (UT) and treated (T) tannery effluent on germination and growth of mung bean seeds. Values are mean ± standard deviation (*n* = 3).

Effluent conc. (%)	Germination (%)		Total length (cm)		Root length (cm)		Shoot length (cm)	
	UT	T	UT	T	UT	T	UT	T
0	100 ± 0	100 ± 0	12.8 ± 1.0	12.8 ± 1.0	2.4 ± 0.8	2.4 ± 0.8	8.9 ± 0.7	8.9 ± 0.7
25	90 ± 10	90 ± 10	6.2 ± 1.6	7.5 ± 1.5	1.6 ± 1.2	3.0 ± 0.0	4.6 ± 0.2	4.7 ± 1.1
50	10 ± 0	90 ± 10	7.2 ± 1.1	7.9 ± 0.6	1.0 ± 0.6	1.9 ± 0.7	4.1 ± 1.5	3.5 ± 0.2
75	0	10 ± 0	0	6.5 ± 0.5	0	1.5 ± 0.1	0	3.5 ± 0.2
100	0	10 ± 0	0	6.0 ± 0.5	0	1.5 ± 0.1	0	3.0 ± 0.2

**Table 3:** The number of bands in control and molecular sizes (base pair, bp) of disappearance (−) and/or appearance (+) of DNA bands in untreated and treated effluent irrigated mung bean plants using Gene tool software.

Primers	Control		Untreated effluent (25%)	Treated effluent (25%)
RPi-C1	4 (283–800 bp)	−	800; 560; 400; 283	800
		+	0	430
RPi-C2	6 (390–1170 bp)	−	970; 390	970; 790; 670
		+	1114; 413	580; 300; 275; 200
RPi-C3	4 (551–842 bp)	−	842; 740; 640; 551	842; 740
		+	0	970;430
RPi-C4	4 (400–930 bp)	−	930; 740; 680	930; 400
		+	0	0
RPi-C5	4 (650–955 bp)	−	955; 850	704; 650
		+	0	0
RPi-C6	4 (172–516 bp)	−	0	516; 244; 172
		+	650; 350; 220	620
RPi-C7	4 (160–1026 bp)	−	1026	0
		+	850; 540; 260; 180	0
RPi-C8	4 (164–510 bp)	−	510; 409	510; 164
		+	750; 620; 350	0

Total	34		18 (−); 12 (+)	15 (−); 8 (+)
